# Fine‐root dynamics vary with soil depth and precipitation in a low‐nutrient tropical forest in the Central Amazonia

**DOI:** 10.1002/pei3.10010

**Published:** 2020-04-22

**Authors:** Amanda L. Cordeiro, Richard J. Norby, Kelly M. Andersen, Oscar Valverde‐Barrantes, Lucia Fuchslueger, Erick Oblitas, Iain P. Hartley, Colleen M. Iversen, Nathan B. Gonçalves, Bruno Takeshi, David M. Lapola, Carlos A. Quesada

**Affiliations:** ^1^ Instituto Nacional de Pesquisas da Amazônia – INPA Manaus Brazil; ^2^ Colorado State University – CSU Fort Collins CO USA; ^3^ Oak Ridge National Laboratory Oak Ridge TN USA; ^4^ Nanyang Technological University – NTU Singapore; ^5^ Florida International University –Miami Miami FL USA; ^6^ University of Antwerp Antwerp Belgium; ^7^ Geography College of Life and Environmental Sciences University of Exeter Exeter UK; ^8^ Michigan State University – MSU East Lansing MI USA; ^9^ University of Campinas – UNICAMP Campinas Brazil

**Keywords:** belowground productivity, fine‐root dynamics, minirhizotrons, precipitation, root turnover, rooting depth, *Terra firme*, tropical forest

## Abstract

- A common assumption in tropical ecology is that root systems respond rapidly to climatic cues but that most of that response is limited to the uppermost layer of the soil, with relatively limited changes in deeper layers. However, this assumption has not been tested directly, preventing models from accurately predicting the response of tropical forests to environmental change.
- We measured seasonal dynamics of fine roots in an upper‐slope plateau in Central Amazonia mature forest using minirhizotrons to 90 cm depth, which were calibrated with fine roots extracted from soil cores.
- Root productivity and mortality in surface soil layers were positively correlated with precipitation, whereas root standing length was greater during the dry periods at the deeper layers. Contrary to historical assumptions, a large fraction of fine‐root standing biomass (46%) and productivity (41%) was found in soil layers deeper than 30 cm. Furthermore, root turnover decreased linearly with soil depth.
- Our findings demonstrate a relationship between fine‐root dynamics and precipitation regimes in Central Amazonia. Our results also emphasize the importance of deeper roots for accurate estimates of primary productivity and the interaction between roots and carbon, water, and nutrients.

## 1 INTRODUCTION

The Amazon rainforest is one of the largest ecosystem carbon (C) reserves in the world, storing approximately 150–200 Pg C in living vegetation biomass and soils (Brienen et al., 2015; Feldpausch et al., 2012). However, there is uncertainty on how this forest will respond to changing climate scenarios and increasing atmospheric CO_2_ concentrations (Cox et al., 2013; Friedlingstein et al., 2006; Lapola, 2018) and drought conditions (Doughty et al., 2015; Lovejoy & Nobre, 2018; Meir, Metcalfe, Costa, & Fisher, 2008). Fine roots (generally defined as roots ≤2 mm in diameter) are a key component of belowground processes, playing an important role in the cycling of water, nutrients, and carbon in terrestrial ecosystems (Jackson, Mooney, & Schulze, 1997; Pregitzer, 2002). Thus, direct observations of tropical forest root dynamics in response to environmental cues are crucial to understand processes underlying current carbon fluxes and to better predict future tropical forest function. Although many studies in the tropics have focused on estimates of aboveground components, there are considerably fewer studies focusing on belowground processes (Clark et al., 2001). Therefore, a first step to improving current and future estimates of tropical forest carbon fluxes is to obtain robust measures of belowground components.

Fine‐root productivity accounts for a significant fraction (22%–40%) of terrestrial net primary production (NPP; Aragão et al., 2009; Bloom, Chapin, & Mooney, 1985; Jackson et al., 1997; McCormack et al., 2015). Furthermore, because fine roots are usually a very dynamic carbon pool and are intimately associated with the surrounding soil, their turnover may constitute a major carbon input to soils and significantly contribute to organic matter accumulation (Lukac, 2012; Rasse, Rumpel, & Dignac, 2005; Russell, Cambardella, Ewel, & Parkin, 2004). Changes in rooting profiles can affect important ecosystem processes such as root chemistry, physiological function, and mycorrhizal colonization leading to shifts in nutrient uptake rate and turnover rates, which might have implications for soil carbon storage (Iversen, 2010; Jackson et al., 1996). Therefore, understanding differences in C allocation to roots and root turnover rates among ecosystems and within the soil depth profiles are crucial to determine the changes in soil C storage and how ecosystems may respond to disturbance and environmental cues (Matamala, Gonzalez‐Meler, Jastrow, Norby, & Schlesinger, 2003; Norby, Fitter, & Jackson, 2000; Vogt et al., 1993).

Soil moisture is a main factor explaining fine‐root dynamics in tropical regions (Cavelier, Wright, & Santamaria, 1999; McGroddy & Silver, 2000; Metcalfe et al., 2008; Yavitt & Wright, 2001) and therefore a possible driver of belowground dynamics under expected climate change scenarios in the tropics (Magrin et al., 2014). For example, a study in seasonal Amazonian forests presented that a decline in soil moisture (from seasonality and experimental drying) decreased root biomass (Metcalfe et al., 2008). In addition, in the middle of the Panama rainfall gradient increased soil moisture in an irrigation experiment increased root productivity (in the dry season) and biomass (Yavitt & Wright, 2001). On the other hand, a study using 48 plots evaluated fine‐root biomass in a rainfall gradient in lowland tropical forests in Panama in different types of soils and found that fine‐root biomass was insensitive to rainfall (Cusack, Markesteijn, Condit, Lewis, & Turner, 2018). These contrasting results indicate that the effect of water availability on root dynamics merit further research in tropical forests.

Some regions of the Amazonia basin already experience seasonal patterns in rainfall with up to 7 months of the year in which evapotranspiration exceeds rainfall (Sombroek, 2001). Deep roots have been hypothesized to enhance plant water availability during the dry season in these areas (Markewitz, Devine, Davidson, Brando, & Nepstad, 2010; Nepstad et al., 1994). However, little is known about how plants from areas that do not experience such severe prolonged drought periods might cope with decreased precipitation or an increase in the length of the dry season (i.e., the Central Amazon; Bonal, Burban, Stahl, Wagner, & Hérault, 2016). It has been hypothesized that plants should allocate more carbon to producing fine roots when and where soil resources (water or nutrients) are limiting in nutrient deficient and seasonally droughted soils (Bloom et al., 1985; Cannell & Dewar, 1994; Chapin, 1980; Thornley, 1972).

In support of resource limitation driving fine‐root processes across tropical landscapes, several studies have found fine‐root standing biomass generally increases with decreasing soil resources availability (Aragão et al., 2009; Espeleta & Clark, 2007; Gower, 1987; Metcalfe et al., 2008; Ostertag, 2001; Powers, Treseder, & Lerdau, 2005). However, measurements of standing stocks alone cannot be used to quantify carbon allocation to roots. Fine‐roots appear to have a greater turnover when there are more soil resources available because fine‐root maintenance costs are greater than construction costs (Aragão et al., 2009; Eissenstat & Yanai, 1997). Thus, even where fine‐root productivity is high, the greater turnover of these roots might lead to lower fine‐root stock. Therefore, in order to understand how much carbon is being allocated to produce fine roots, it is necessary to measure not just fine‐root standing biomass, but also productivity and turnover.

In particular, in Central Amazonia, where there is low phosphorus (P) and base cation availability in the mineral soil, fine‐root standing stock and fine‐root productivity have been shown to decrease with soil depth (Klinge, 1973; Noguchi et al., 2014; Quesada et al., 2011; Silver et al., 2000). A proliferation of fine roots near the soil surface allows roots to intercept nutrients from decomposing leaf litter, before they reach the mineral soil, allowing the direct cycling of nutrients, which is likely an adaptation to the low nutrient supply in infertile soils (Jordan, 1985; Richards, 1996; Sayer, Tanner, & Cheesman, 2006; Stark & Jordan, 1978; Went & Stark, 1968). This shallower rooting depth is likely a central strategy of roots to cope with soil nutrient limitation, but might make them more vulnerable to drying (Yavitt & Wright, 2001). Therefore, deeper roots could play an important role in water uptake and nutrient cycling during the dry season when surface soils have less water available.

Despite the importance of fine roots for biogeochemical processes, most fine‐root dynamics studies, particularly in tropical forests, have been focused on the top 10–30 cm of the soil profile. Thus, there is still a lack of studies of fine‐root dynamics across tropical forests, mainly on deeper layers (Iversen et al., 2017). Methodological difficulties in assessing belowground compartments, particularly in remote tropical sites and clay‐rich soils, have hindered progress in measuring fine‐root dynamics (Judd, Jackson, & Fonteno, 2015). For long‐term monitoring studies, minirhizotrons are a preferred method to quantify root dynamics because each root can be tracked over time with minimal disturbance, which contributes to a more trustworthy measurement of root productivity and mortality across a range of depths along the soil profile (Hendrick & Pregitzer, 1996b; Rewald & Ephrath, 2013). One disadvantage of minirhizotron methodology, however, is that the roots analyzed cannot be collected; therefore, root biomass cannot be measured directly. Nevertheless, root diameter and length can be evaluated over time and biomass can be estimated (Iversen, Ledford, & Norby, 2008).

In this study we provide novel, high spatial and temporal resolution estimates for fine‐root dynamics using minirhizotrons in an Amazonian plateau tropical forest. We quantified the patterns and controls of fine‐root productivity, standing stock, and mortality across the vertical soil profile, and we calculated fine‐root population turnover with soil depth. This research was conducted at a Central Amazonian *terra firme* forest site that is representative of the most extensive soil type across the Amazonia basin. The specific objectives of this study were to: (a) quantify fine‐root dynamics over the year identifying seasonal patterns and what factors might be affecting it; and (b) quantify fine‐root dynamics throughout the vertical soil profile to a depth of 90 cm. Based on responses that have been observed in both tropical and temperate forests, we hypothesized that root standing biomass would decrease with soil depth, but that roots at depth would turnover more slowly (i.e., have a longer lifespan). Furthermore, we hypothesized that root production would be greatest during the wet season, and mortality greatest during the dry season and that fine‐roots at surface layers would respond more to seasonal environmental changes.

## 2 MATERIAL AND METHODS

### 2.1 Site description

The study site is in an old‐growth, Lowland Dense Ombrophylous Terra Firme forest (IBGE, 2012) located on a plateau in the Cuieiras Biological Reserve – ZF2, AM, Brazil (2°35′40″S, 60°12′28″W). This study is part of the baseline pretreatment research that has been conducted in two Amazon FACE (Free‐Air CO_2_ Enrichment Research Program in the Amazon forest) pilot plots prior to the imitation of CO_2_ treatments; these plots are 30 m in diameter and separated by approximately 90 m (Fleischer et al., 2019; Lapola, 2018; Lapola & Norby, 2014; Norby et al., 2016). These plots were used as blocks in our experiment design. The mean annual temperature is 26°C (Araujo et al., 2002), and the mean annual precipitation ranges between 1,900 and 2,400 mm, with a dry season from July to October (Lovejoy & Bierregaard, 1990). The soil type is a Geric Ferralsol (Alumic, Hyperdystric, Clayic; Quesada et al., 2010, 2011), which is highly weathered and nutrient depleted, with particularly low concentrations of rock‐derived elements (Quesada et al., 2010, 2011). Soil surface (0–15 cm) total carbon (C) ranges spatially from 2.9% to 3.6%, total nitrogen (N) from 0.2% to 0.3%, total phosphorus (P) from 50 to 130 mg/kg, and the sum of exchangeable bases ranges from 0.12 to 0.25 cmol_c_/kg. Soil characteristics of the top 100 cm of the soil in the north‒south transect adjacent to our plots can be found at the Table S1. The water table is usually located at a depth between 30 and 35 m (Tomasella et al., 2010), and median canopy height is 30 m with emergent trees over 45 m tall (Vieira et al., 2004). In the two 30‐m diameter plots where we studied fine‐root dynamics, there were at least 293 different species and 40 different families of trees >2 cm diameter at breast height (dbh) and 55 trees with dbh ≥ 10 cm. On average, tree basal area was 37 cm^2^/m^2^, maximum height was 36 m, and leaf area index was 5.3 (Pereira et al., 2019). More information about our site can be found in Pereira et al. (2019) and Fleischer et al. (2019).

### 2.2 Root standing stock, productivity, mortality, and turnover

Fine‐root dynamics were measured using minirhizotrons (Bates, 1937; Johnson, Tingey, Phillips, & Storm, 2001). In December 2014, five acrylic minirhizotron tubes with 5 cm inner diameter and 2 m length were installed at an approximately 45° angle by using a hand auger in each of the two plots (adapted from Norby, Ledford, Reilly, Miller, & O'Neill, 2004). Tubes were at least 1 m apart, and insertion angles were measured during and after installation. The aboveground portion of the tubes was capped and covered with black foam and a PVC tube to exclude light and protect from damage from animals. Images were recorded monthly from November 2016—2 years after tube installation allowing the root system to stabilize after disturbance—to December 2017. Images were collected with a BTC‐2 minirhizotron camera (Bartz Technology) at each of the 90 viewing windows (10.64 mm × 14.23 mm) of 96 dpi each, with a total viewing area of 0.0136 m^2^. Monthly pictures were taken once or twice at the same position along each tube to observe root dynamics along the soil profile over time. One tube was removed from further analysis because clay particles smeared on the tube surface led to blurred images (*n* = 5 in plot 1 and *n* = 4 in plot 2).

We did not collect images in May and June 2017 due to equipment failure. A total of 14,580 images were analyzed in the remaining 9 tubes over 18 collection periods. All images were analyzed by the same person with Rootfly software (http://www.plant‐image‐analysis.org/software/rootfly). The length and diameter of each fine‐root segment (only roots less than 2 mm in diameter) were measured, and the incremental growth, death, or disappearance was recorded. Root length production per minirhizotron window area per day (mm m^−2^ day^−1^) and standing crop (m/m^2^) were calculated for each sample date. Root mortality (mm m^−2^ day^−1^) was considered when the entire root or a part of the root disappeared from one date to the other. Roots were not considered dead until they disappeared, so we conservatively equate “disappearance” with “mortality.” Fine‐root turnover (year^−1^) was calculated as fine‐root mortality divided by peak root standing crop which is the date of the maximum standing stock (Iversen et al., 2008; Satomura, Fukuzawa, & Horikoshi, 2007) using population mean values. Root standing stock, productivity, mortality, and turnover were aggregated by depth intervals (every 10 cm or 30 cm) to 90 cm. Root diameter classes were separated into 0.5 mm size classes between 0 and 2.0 mm for all variables. To examine the relationship between fine‐root dynamics and precipitation, we used monthly precipitation for our study months collected at the K34 tower, which is located 2 km away from our site. Precipitation measured at the site was nearly identical to that measured at the tower 2 km away, but because of gaps in the site data, we used the more complete record from the tower.

### 2.3 Estimation of root biomass

Minirhizotron‐based fine‐root dynamics values are initially reported as length per window area (m/m^2^). We estimated biomass by finding an allometric relationship between root mass, diameter, and length (see Section 3) from fine roots at our site, following the approach described by Iversen et al. (2008). Roots were collected in areas along transects adjacent to the AmazonFACE plots to avoid disturbance within long‐term monitoring areas. Root samples were collected from 18 soil cores with a diameter of 10.5 cm and a depth of 15 cm in February, April, August, and November 2016. A subset of roots was randomly selected from each soil core, with roots being cleaned and scanned individually with WinRHIZO™ (Regent Instruments Inc.) to obtain length and diameter for each root (*n* = 543). Roots were dried at 65°C for 72 hr or to constant weight and weighed. For each scanned root image, diameter, length, and mass were used to find a relationship between root mass per unit length (RML; mg/cm) and diameter (mm) for each root. We tested several linear and non‐linear regressions to determine the best‐fitted model to our data and the Akaike information criterion (AIC) was used to determine the best model. The equation relating RML and diameter was then used to estimate root biomass from minirhizotron images. The biomass of roots in each minirhizotron image was scaled to a volume of soil using the depth‐of‐field (DOF) approach; we assumed that the DOF in each minirhizotron window was 2.5 mm (Johnson et al., 2001). The DOF was calculated by calibrating minirhizotron estimated mass with root mass harvested from 18 soil cores near the minirhizotron tubes for the same depth (0–15 cm), diameter cutoff from the cores (<1 mm) and date. We assumed that DOF and RML versus diameter did not vary with depth. We used a diameter cutoff of <1 mm because roots from cores were separated in <1 mm and >1 mm previously. To convert fine‐root productivity data from Mg/ha to Mg C/ha, we used the percentage of C in roots, which was 43.85% (± 0.27 SE; *n* = 66) for roots in the 0–15 cm depth (Fuchslueger, unpublished data).

### 2.4 Statistical analyses

All data were analyzed in R software (R Core Team, 2017). Fine‐root dynamics (productivity, mortality, standing stock) from minirhizotron images were analyzed using linear mixed models to control for the hierarchical experimental design. We performed separate models testing whether and how (a) season, (b) depth interval, and (c) diameter class (fixed factors) influenced fine‐root (<2 mm; a) productivity, (b) mortality, and (c) standing stock (dependent variables) for each dependent variable. Season, depth interval, and diameter class (fixed factors) were nested within tube, and tubes were nested within blocks (random effects). Seasons were separated into wet and dry season, where January, February, and March 2017 represent the wet season (average precipitation = 328 mm) and July, August, and September 2017 represent the dry season (average precipitation = 89 mm). Diameter classes were separated into 0–0.5, 0.5–1, 1–1.5, and 1.5–2 mm increments and depth intervals were separated in 30 cm intervals (0–30, 30–60, and 60–90 cm).

We also analyzed whether and how different depth intervals and diameter classes (fixed factors) influenced fine‐root turnover (dependent variable) by testing different mixed effects models separately for each fixed factor. Fine‐root diameter class (fixed factor) was separated by 0.5 mm increments from 0 to 2 mm (as above) where total fine‐root turnover in different diameter classes is nested within tube, and tubes are nested within blocks (random effects). To analyze depth intervals as the fixed effect, we considered fine‐root turnover as the sum of mortality divided by the sum of fine‐root standing stock from all tubes in each block. Here, we only considered roots <1 mm diameter to control for the bias introduced by the few records of roots >1 mm since for some depth intervals there was stock, but no mortality for roots >1 mm which would underestimate root turnover if considered. Ultimately, depth intervals were separated in 30‐cm depth increments and were nested within block (random effect). To produce a finer spatial analysis of root turnover with soil depth, we also analyzed it by 10 cm depth intervals from 0 to 90 cm. In this case we considered fine‐root turnover from all tubes (*n* = 9) as the average of mortality divided by the peak of fine‐root standing stock which is the maximum value for standing stock during the collection period and calculated the standard error of the ratio according to Seltman (2019) for each depth interval. We performed a regression test and chose the model that best fitted our data, by identifying the one with the lowest AIC when comparing linear and non‐linear (quadratic, power, exponential) functions.

To examine the effect of precipitation at different depth intervals (fixed factors) on fine‐root dynamics, the dependent variables (productivity, mortality, and root standing stock) were calculated as the average from all the tubes (*n* = 9) for each depth interval and same month as the precipitation data. To control for the temporal pseudo‐replication of the experimental design, we included time as a random effect and used an autoregressive (1) variance structure (corAR1) to account for the time series autocorrelation inherent in the data.

For all the linear mixed model analyses, we used the R packages “NLME” (Pinheiro, Bates, DebRoy, & Sarkar, 2020) and “LME4” (Bates, Maechler, Bolker, & Walker, 2015). We tested the assumptions for normality and response variables were log‐transformed as needed to meet assumptions for linear models. We tested model fits using diagnostic plots and used weights to control for heteroscedasticity among factor levels when necessary (Pinheiro & Bates, 2000). We choose the best models based on the AIC. Post hoc Tukey tests were performed to examine the differences between least square means of each fixed factor. Although replication was low, we found that randomly removing up to three tubes from the data set, made no difference in the results. Data and the R code from this study are publically available (Cordeiro et al., 2019). Table S2 provides statistical information.

## 3 RESULTS

**Figure 1:**
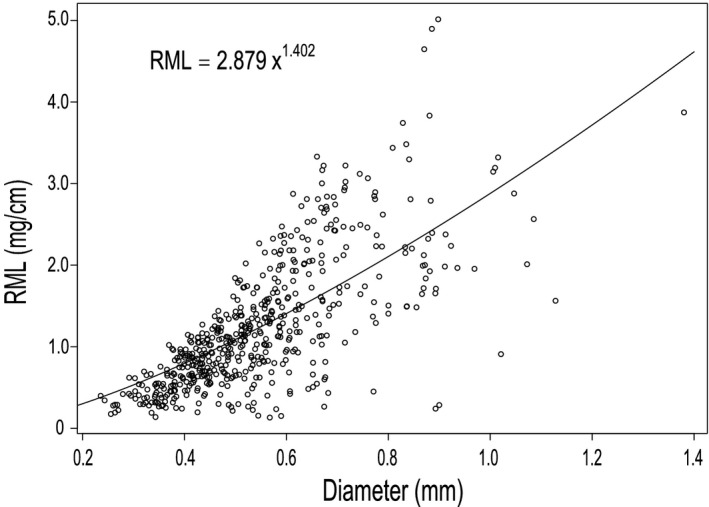
Relationship between root mass per unit root length (RML, mg/cm) and root diameter (mm) from soil cores near AmazonFACE plots; *n* = 543; *r^2^* = .51; *p* < .0001

Annual fine‐root length productivity and mortality per image window area (0–90 cm depth) were 23.0 ± 3.3 and 12.6 ± 2.6 m m^−2^ year^−1^, respectively, and peak fine‐root standing length was 46.6 ± 5.0 m/m^2^ (Table 1). Total fine‐root population turnover was 0.27 year^−1^ (i.e., the average fine‐root population lifespan across all root sizes and soil layers was 3.7 years). The relationship between root diameter and RML followed a positive power function, where RML = 2.879 × Diameter^1.402^ (Figure 1, *r* ^2^ = .51). We used this equation to scale up to total root standing biomass and biomass productivity from minirhizotron surface area to ground area. Total fine‐root biomass (0–90 cm depth) was estimated at 13.1 ± 1.9 Mg/ha (5.7 ± 0.9 Mg C/ha) and productivity at 6.3 ± 0.8 Mg ha^−1^ year^−1^ (2.8 ± 0.4 Mg C ha^−1^ year^−1^).

**Table 1:** Fine‐root length, productivity, mortality, and turnover (mortality/standing stock) in relation to depth in the soil profile

Depth interval (cm)	Standing stock (m/m^2^)	Productivity (m m^−2^ year^−1^)	Mortality (m m^−2^ year^−1^)	Turnover (year^−1^)
0–30	25.1 ± 4.0	13.7 ± 2.2	8.4 ± 2.0	0.33
30–60	12.1 ± 2.8	5.8 ± 1.5	3.4 ± 1.2	0.28
60–90	9.3 ± 1.8	3.5 ± 1.0	0.8 ± 0.3	0.09
SUM (0–90)	46.6 ± 5.0	23.0 ± 3.3	12.6 ± 2.6	0.27

Note

Values for root length standing stock at the peak standing stock date, productivity, mortality per unit minirhizotron window area are the average of nine minirhizotron tubes ± *SE*. Turnover rates were calculated as fine‐root mortality divided by standing stock.

### 3.1 Diameter and depth distributions

**Figure 2:**
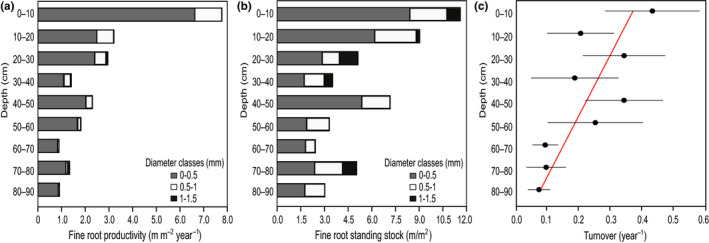
Fine‐root dynamics by diameter classes and depth distribution at AmazonFACE site using minirhizotron; (a) fraction of annual root productivity by depth and diameter class; (b) fraction of fine‐root standing stock at its peak by depth and diameter class and (c) fine‐root turnover (fine‐root mortality divided by fine‐root peak standing stock) by depth for the 0–1 mm diameter class (*r* ^2^ = .62; *p < *.01; Turnover (year^−1^) = −0.0037 * Depth (cm) + 0.41). Data are means ± *SE* of the ratio estimated according to Seltman (2019)

Root diameter had a range of 0.099–1.55 mm, so all roots observed in the minirhizotrons are considered fine roots. Root standing stock (*F*_3,11_ = 41.78, *p < *.001), productivity (*F*_3,10_ = 23.33, *p < *.001), mortality (*F*_3,10_ = 9.19, *p < *.004), and turnover (*F*_2,10_ = 9.41, *p < *.001) differed among diameter classes (0–0.5, 0.5–1, 1–1.5, 1.5–2 mm). Smaller diameter roots (0–0.5 mm) made up the greatest proportion (76%) of fine‐root standing stocks at the peak standing stock date (Figure 2a). A similar pattern was observed for root productivity where 82.2% of fine‐root productivity was observed in the smallest diameter class (Figure 2b) and for root mortality where 85% was observed in the smallest diameter class. The highest fine‐root turnover (0.33 ± 0.08) was observed in the smallest diameter class (0–0.5 mm), decreasing to 0.29 ± 0.12 at the 0.5–1 mm diameter class and to 0.01 ± 0.01 at the 1–1.5 mm diameter class.

Root standing length varied with depth (*F*_2,16_ = 6.88, *p < *.008), with over half of fine‐root standing length (53.9%) observed in the 0–30 cm soil layer, with the remainder (46.1%) observed in the 30–90 cm layer (Figure 2a). Fine‐root length productivity also differed among depths intervals (*F*_2,16_ = 12.93, *p < *.001) where, 59.4% of fine‐root production occurred in the 0–30 cm depth interval, and 40.6% of root productivity occurred in the 30–90 cm depth (Figure 2b). Fine‐root mortality also varied with depths intervals (*F*_2,16_ = 9.19, *p < *.003) with greater mortality in surface soil layers. Fine‐root turnover (0–2 mm diameter class) decreased significantly with depth for the 30 cm depth increments (*F*_2,14_ = 4.86, *p < *.003) and also decreased linearly with depth for the 10 cm depth increments (*r* ^2^ = .62; *p < *.01; Turnover (year^−1^) = −0.0037 * Depth (cm) + 0.41; Figure 2c).

### 3.2 Seasonal differences in fine‐root standing biomass and productivity

**Figure 3:**
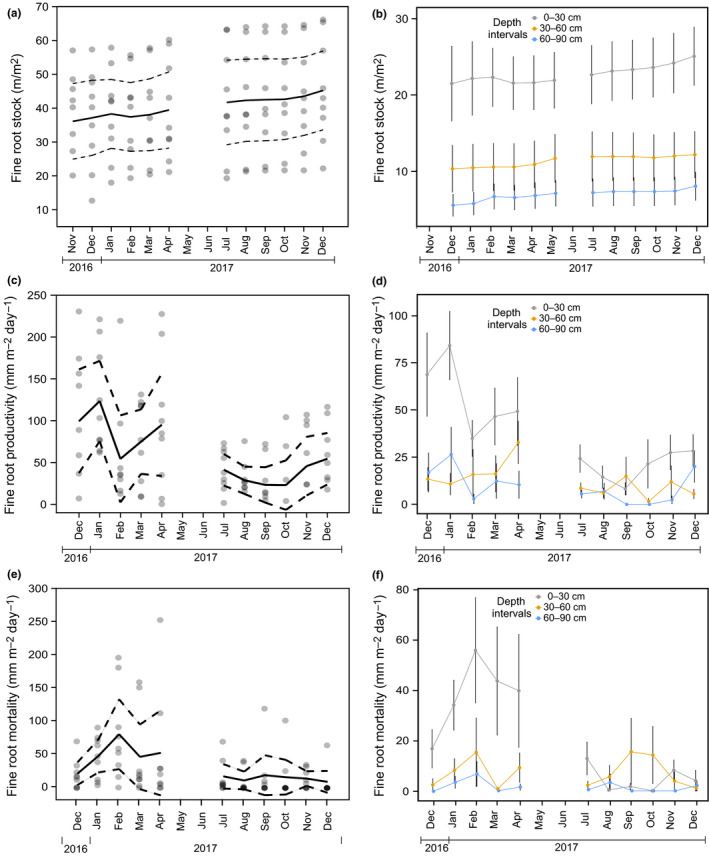
Fine‐root dynamics in the AmazonFACE site; for (a, c, e) gray symbols represent each of the nine tubes with solid and dashed lines representing means ± 95% CI (confidence interval). For (b, d, f), dots represent fine‐root stock, productivity, or mortality means ± *SE* for each date and colors represent each depth interval (0–30, 30–60, and 60–90 cm). (a) Seasonal pattern of total fine‐root standing stock; (b) seasonal pattern of fine‐root standing stock in three‐depth intervals; (c) seasonal pattern of total fine‐root productivity; (d) seasonal pattern of fine‐root productivity divided by depth intervals; (e) seasonal pattern of total fine‐root mortality; (f) seasonal pattern of fine‐root mortality divided by depth intervals

There was no significant difference in fine‐root standing length (*F*_1,8_ = 4.76, *p > *.06) between seasons, although the fine‐root standing length was slightly higher (1.08 times) in the dry season when compared to the wet season (Figure 3a). Greater root stock lengths were consistently closer to the soil surface over the year (Figure 3b). The minimum root standing length of 36.1 ± 4.8 m/m^2^ was observed in November 2016 and increased to the maximum root standing length of 46.6 ± 5.0 m/m^2^ which was observed in December 2017 (Figure 3a). In contrast, fine‐root length productivity varied significantly between seasons (*F*_1,8_ = 27.69, *p < *.001) with productivity 5.37 times higher in the wet season than in the dry season. Minimum and maximum productivity rates were 15.1 ± 6.1 and 142.8 ± 5.5 mm m^−2^ day^−1^ occurring in October and January 2017, respectively (Figure 3c) with the highest peaks in productivity occurring between December 2016 and January 2017 at the 0–30 cm depth layer (Figure 3d). Fine‐root mortality also varied between seasons (*F*_1,8_ = 12.00, *p < *.009) where mortality was 44.8 times higher in the wet season. The minimum mortality occurred in December 2017 (7.2 ± 5.2 mm m^−2^ day^−1^) and maximum in February 2017 (79.2 ± 22.9 mm m^−2^ day^−1^; Figure 3e) and the peak of mortality occurred between January and February 2017 at the 0–30 cm depth layer (Figure 3f).

**Figure 4:**
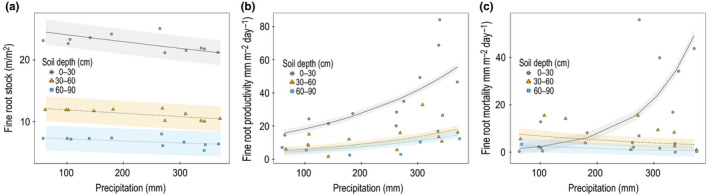
Models of fine‐root dynamics with confidence interval associated in the AmazonFACE site for the 0–90 cm depth interval by 30 cm intervals increments at different average month precipitation (mm) where *a* = precipitation (mm). For the 0–30 cm depth equation, *b* = 0 and *c* = 0; for the 30–60 cm depth equation, *b* = 1 and *c* = 0; and for the 60–90 cm depth equation, *b* = 0 and *c* = 1. Precipitation data are from the nearby K‐34 tower. (a) Total fine‐root stock (m/m^2^) = exp (3.22 − 0.0004*a − 0.7*b − 1.2*c); (b) total fine‐root productivity (mm m^−2^ day^−1^) = exp (2.49 + 0.004*a − 1.12*b − 1.32*c); (c) total fine‐root mortality (mm m^−2^ day^−1^) = exp (−0.26 + 0.011*a + 2.46*b + 1.54*c − 0.014*a*b* − 0.016*a*c)

Root length dynamics throughout the year varied with patterns in precipitation (Figure 4). Although fine‐root standing length was not different between seasons, it was negatively related to precipitation (*F*_1,9_ = 7.09, *p* < .03, *r* ^2^ = .98) when depth intervals (30 cm increments) were considered in the model (Figure 4a). This relationship was more accentuated at the 0–30 cm depth. In contrast, fine‐root productivity increased with increasing precipitation in all depth intervals (*F*_1,9_ = 25.06, *p* < .001, *r* ^2^ = .61) and this relationship was even stronger in the 0–30 cm layer (Figure 4b). For fine‐root mortality, there was an interaction between precipitation and depth intervals (*F*_2,14_ = 6.47, *p* < .02, *r* ^2^ = .64), where mortality at the 0–30 cm depth increased with increasing precipitation, but in deeper layers, decreased slightly with increasing precipitation (30–60 and 60–90 cm; Figure 4c). Therefore, fine‐root productivity and mortality in the surface layer (0–30 cm) show an opposite seasonal pattern compared to fine‐root standing stocks, with root productivity and mortality peaking when standing stocks are low (when there is more precipitation).

## 4 DISCUSSION

While it is easy to appreciate the grandeur and complexity of the towering trees overhead in a mature tropical forest, a keen observer might notice the network of fine roots creeping across the surface of the soil (Norby, 2017). What remains unseen is the network of fine roots below the surface—a dynamic and spatially complex ecosystem that is critical to the functioning of the forest. Using minirhizotrons to see below the surface in a mature forest in Central Amazonia, we demonstrated the importance of measuring fine roots throughout the soil column.

Our direct observations of fine‐root dynamics to a depth of 90 cm enabled us to reach three important advances in our understanding of fine‐root dynamics in this site in a tropical forest: (a) Although the largest fraction of fine‐root biomass and productivity is in the top 10 cm of the soil profile, a substantial fraction is deeper than 30 cm (46.1% and 40.6%, respectively); (b) As is often assumed but rarely observed, fine‐root turnover declined with depth; (c) Seasonal variation in precipitation drives root dynamics, but the direction and strength of the influence of precipitation varies with depth. Our data extend the quantification of root dynamics to deeper in the soil profile than previous studies in tropical forests, contributing to our understanding of ecosystem NPP, carbon cycling, and environmental controls on fine‐root dynamics.

### 4.1 Fine‐root characterization and NPP across the soil profile

Our results indicate that most of fine‐root biomass and productivity is in the top 10 cm of the soil profile. However, a substantial fraction (>40%) is deeper than 30 cm and would be missed in most studies of tropical roots. Vertical fine‐root distribution was modeled by the equation (Gale & Grigal, 1987): *Y* = 1 − *β^D^*, where *D* is depth (cm), *Y* is the proportion of roots from the surface to depth *D* and *β* is a numerical index of rooting distribution. We calculated a *β = *0.974, estimating that 96.9% of fine‐root biomass can be found at the 90 cm layer. The index of root distribution was greater than the average estimate for tropical evergreen forests indicating greater proportion of roots with depth (Jackson et al., 1996). The standing stock of root biomass of 13.1 Mg/ha (5.7 Mg C/ha) at 90 cm in this study is greater than other measurements in the region. For example, Klinge (1973) measured fine‐root biomass to approximately 1 m depth as 8.4 Mg/ha, and Trumbore et al. (2006) reported 3.8 Mg C/ha from 0 to 6 m which is a much lower value when compared to the other studies. This discrepancy might be a consequence of the differences in methodologies as that study estimated root mass from cores using a flotation method that may have missed many of the smaller diameter and living roots (Böhm, 1979) or location since it was conducted in eastern Amazonia. In contrast, another study measured root biomass (<2 mm) to 40 cm depth from a similar site in Central Amazonia and found a value of 8.7 Mg/ha (Noguchi et al., 2014). This value is actually very similar to the biomass we observed in the top 40 cm, but our data suggest that by sampling to only 40 cm Noguchi et al. (2014) may have missed at least 40% of the total fine‐root biomass. Another factor that deserves attention and future research is the almost double of fine‐root stock of smallest diameter roots (0–0.5 mm) on the 40–50 cm depth when compared to the 30–40 cm depth.

Fine‐root productivity throughout the soil profile for the Amazon rainforest is rarely directly measured. Rather, root productivity below 30 cm is generally estimated, ranging between 2 and 7.6 Mg C ha^−1^ year^−1^ for the 1 m depth (Aragão et al., 2009; Malhi et al., 2009). These estimates were obtained from direct measurements to 20–30 cm depth using different methods, such as ingrowth and sequential cores (Aragão et al., 2009; Jiménez, Moreno, Nuela, Patiño, & Lloyd, 2009). Then, fine‐root productivity was extrapolated to 1 m by assuming that fine‐root productivity per unit fine‐root biomass and fine‐root biomass per unit coarse‐root biomass are invariant with depth (Malhi et al., 2009); a coarse‐root biomass profile (Fisher et al., 2007) was used to estimate scaling factors. There are no published measurements of fine‐root productivity for the Manaus region, however, it was estimated from an average between other similar sites to be 3.3 or 2.1 ± 0.7 Mg C ha^−1^ year^−1^ (Aragão et al., 2009; Malhi et al., 2009). Despite differences in root measurement methodology, assumed carbon fraction, and the many scaling assumptions, these two estimates fortuitously bracket our more direct measurement of fine‐root productivity to 90 cm (2.8 ± 0.4 Mg C ha^−1^ year^−1^). Direct measurements of fine‐root productivity in deeper layers are extremely important to produce improved estimates of fine‐root contribution to total NPP. Net primary productivity of this forest has been estimated to be 10.1 or 11.4 Mg C ha^−1^ year^−1^ (Aragão et al., 2009; Malhi et al., 2009). Hence, fine‐root productivity to 90 cm soil depth represents about 26% of NPP. Although there is a decrease in fine‐root productivity with depth, the fraction 30–90 cm contributes over 12% to total NPP in this forest and would be unaccounted for if it has not been measured.

We hypothesize that the decrease in fine‐root standing stock and productivity with soil depth in our study is related to soil resource availability. Many of the soils in the tropics are strongly nutrient limited, particularly for rock‐derived nutrients such as P (Quesada et al., 2010, 2012; Walker & Syers, 1976). Plants can obtain nutrients as soon as organically bound nutrients from the litterfall are mineralized (Jordan, 1985; Richards, 1996; Sayer et al., 2006; Vitousek, 1982, 1984) or even directly from the decaying material through interactions with mycorrhizas and exudation of extra‐cellular compounds (Went & Stark, 1968). Therefore, most of root biomass is located at the top 10 cm layer (Figure 2) as an adaptation to maximize nutrient uptake. In our study, most of root length stock (93%) and length productivity (99%) occurs in the finest roots (<1 mm) and in the uppermost layers. Thinner roots are able to explore larger soil volumes and may take up resources without investing as much carbon into biomass and structure, which can also be a strategy to uptake resources in a limiting environment (McCormack et al., 2015).

Finally, it is also important to highlight that there is no consensus on the definition of “deep roots” in the literature. Maeght, Rewald, & Pierret (2013), for example, proposed that deep roots would belong to depths below 1 m based on global median depth of root profiles of 0.88 m (Schenk & Jackson, 2002). On the other hand, this definition can be more challenging considering the global diversity of systems and species (Pierret et al., 2016). Our study does not measure fine‐root biomass and dynamics below 1 m but, to our knowledge, it is the first one to directly measure fine‐root dynamics over time below 30 cm in Central Amazonia. Future studies considering deeper roots dynamics (>1 m) would be important, since roots were found in depths of >15 and 11–18 m in the northeastern and eastern Amazonia respectively (Davidson et al., 2011; Nepstad et al., 1994) and to 4 m in central Amazonia (C.A. Quesada, pers. comm.).

### 4.2 Fine‐root turnover in tropical forests and across depth

Fine‐root turnover is an important contributor to resource cycling in ecosystems. The value for fine‐root turnover we established (0.27 year^−1^) is lower than might be expected, but it is within the range reported for other Amazonian forests (0.14–1.3 year^−1^; Aragão et al., 2009; Graefe, Hertel, & Leuschner, 2008; Jiménez et al., 2009; Trumbore et al., 2006). Variation in fine‐root turnover throughout the Amazonia basin can be related to soil nutrient content (Aragão et al., 2009). One reason for that is that root maintenance costs can be greater than construction costs for new roots when soil nutrient resources are plentiful (Eissenstat & Yanai, 1997). Our low turnover value compared to other tropical systems could be related to the low P and base cations content in our site. Nevertheless, direct comparison between studies is somewhat difficult since there are many methodological variations between them (Hendricks et al., 2006). If we consider fine‐root turnover just for the 0–30 cm depth, as in most of the studies throughout Amazonian forests, this value increases to 0.33 year^−1^. Therefore, our low fine‐root turnover could be also a consequence of measuring soil layers deeper than 30 cm where roots have lower turnover. In addition, if we consider fine‐root turnover as productivity (instead of mortality) divided by stock, it increases to 0.49 for the 0–90 cm depth which is an even higher value, but still lower than all the turnover values calculated in the same way throughout the Amazonia basin from Aragão et al. (2009). Additional minirhizotron‐based studies and in vivo comparisons between methods are important endeavors to tackle in the future (McCormack et al., 2015).

Our documentation of a linear decline in fine‐root turnover is a key result to understand how C stocks can change in response to different environmental conditions. Although there are a few observations in temperate forests suggesting that turnover tends to decrease with soil depth (Baddeley & Watson, 2005; Gaudinski et al., 2001; Germon et al., 2015; Hendrick & Pregitzer, 1996a; Joslin, Gaudiniski, Torn, Riley, & Hanson, 2006), comparable data from the tropics are mostly lacking. In the Amazon, Trumbore et al. (2006) reported no relationship between turnover and depth by using assumptions based on root decomposition rates. However, using direct measurements in the present study, we found a significant linear decline in fine‐root turnover with depth, which should be more accurate than the previous estimates. The slower turnover in deeper layers can be related to the low amount and activity of soil microorganisms (Taylor, Wilson, Mills, & Burns, 2002) and also to the lower nutrient concentrations in deeper layers on this type of soil (Table S1) leading to a lower decomposability (Pregitzer, Laskowski, Burton, Lessard, & Zak, 1998). Thus, changes in root depth distribution can potentially lead to changes in soil carbon storage and C fluxes both from live roots, particulate organic carbon or in stabilized soil organic matter (Rasse et al., 2005). Although our results show a strong effect of depth on fine‐root turnover, turnover can also be influenced by many other factors such as microsite conditions, root development patterns, growing season length, plant mineral nutrient conservation, mycorrhizal colonization, root maintenance respiration, and specific root length (Eissenstat, Wells, Yanai, & Whitbeck, 2000; Gill & Jackson, 2000; Majdi, Pregitzer, Moren, Nylund, & Agren, 2005). In addition, 1 year of observation may be insufficient to capture an equilibrium value of turnover (Iversen et al., 2008). Identifying patterns and variation in fine‐root dynamics across ecosystems using the same methodology and for a longer time represents a basic and necessary step to improve understanding of plant and ecosystem processes.

### 4.3 Seasonal variation in root dynamics

Fine‐root dynamics was influenced by rainfall patterns with greater productivity and mortality in the rainy season, mainly in the surface soil, which is consistent with patterns observed in other tropical regions (Cavelier et al., 1999; Green, Dawson, Proctor, Duff, & Elston, 2005; Rodtassana & Tanner, 2018; Yavitt & Wright, 2001) and in the Amazonia basin (Girardin et al., 2016; Metcalfe et al., 2008). The higher root growth with precipitation can be related to both physical and chemical properties of the soil. The type of soil in the present study presents a high concentration of macropores at depths to 80 cm, which favors rapid water drainage and low water retention leading to a seasonal variation in water content with less soil moisture near the surface at the end of the dry season (Broedel, Tomasella, Cândido, & Randow, 2017; Tomasella & Hodnett, 1996). As a consequence, root turgor pressure can decrease in a dry soil, which can impede root growth in a denser soil (Bengough et al., 2006; Tormena, Silva, & Libardi, 1999).

An increase in soil water content can influence soil nutrient availability and soil microbial dynamics, which can in turn have consequences on root dynamics. Peak litterfall biomass in Central Amazonia occurs during the dry season (Wu et al., 2016), but peak decomposition of litterfall occurs at the onset of the wet season when there is enough moisture for decomposition by microorganisms, with water availability playing an important role in mobilizing nutrients (Buscardo et al., 2018; Luizão & Schubart, 1987). Therefore, the greater fine‐root productivity in the wetter months at the surface layers may be a response to gain access to nutrients derived from litterfall decomposition since roots usually proliferate when water and nutrients are more available (Hodge, 2004; Prior, Rogers, Mullins, & Runion, 2003). Also, the greater mortality of roots is probably related to the highest abundance of decomposers during the rainy season (Buscardo et al., 2018; Luizão & Schubart, 1987).

Although greater fine‐root growth at surface layers in the wet season has been already reported for the Amazon (Luizão, Luizão, & Proctor, 2007; Rodtassana & Tanner, 2018), our study provides new information about root growth in deeper soil layers to changes in precipitation. Some studies have indicated that during the dry season, root growth increases in deeper soil layers to absorb water in these layers (Dickmann, Nguyen, & Pregitzer, 1996; Hendrick & Pregitzer, 1996a; Joslin, Wolfe, & Hanson, 2000; Nepstad et al., 1994; Sommer, Denich, & Vlek, 2000; Vasconcelos, Casagrande, Perecin, Jorge, & Landell, 2003). In contrast, we observed a slight increase in root growth in deeper layers when precipitation was higher (i.e., during the wet season, not the dry season). The soil type in the present study has high soil water content and low variation between seasons from 80 to 480 cm of soil depth compared to the 0–80 cm layer as a consequence of an increase in total porosity and the presence of microaggregates and microfissures among the microaggregates (Broedel et al., 2017). Therefore, fine roots at deeper layers might not be as limited by water in the dry season as the roots at surface soil layers.

In deeper soil layers, fine roots do not have the same access to high quantities of nutrients released by litterfall decomposition as in the surface layers. However, the slight increase in fine roots in deeper layers in the wet season could be related to the greater access to nutrients leached from surface to deeper soil layers when precipitation is higher (Melgar, Smyth, Sanchez, & Cravo, 1992). Interestingly, periods of increased root production and high stocks are not the same. It appears that, even with the lower soil water variation between seasons at deeper layers, plants still need to maintain a higher fine‐root stock at these layers during the drier months. This response can be an important strategy to redistribute water to the whole root system and to surface soil (hydraulic redistribution) keeping them less dry avoiding dissection and mortality of existent roots and microbes (Oliveira, Dawson, Burgess, & Nepstad, 2005).

Peak root growth comes at the expense of carbon used for aboveground growth, thus representing a trade‐off between competing plant sinks (Comas, Anderson, Dunst, Lakso, & Eissenstat, 2005). In Amazonia, peak leaf production occurs when root production is at its minimum, indicating temporal resource partitioning in carbon allocation to the canopy and root production (Girardin et al., 2016). Despite higher wood production in the wet season, the percentage of carbon allocation to wood productivity over the year appears to be constant in humid plots along the Amazonia basin (Girardin et al., 2016; Ourique et al., 2016). Thus, carbon costs of peak root growth may be timed to balance carbohydrate availability with periods of high nutrient and water availability, maximizing resource acquisition and supporting wood growth. However, further studies will be needed to better understand the mechanisms driving this trade‐off in plant allocation which should also include measurements of plant respiration, endogenous factors, microbes and whether this pattern is persistent across tropical forests.

## 5 CONCLUSION

Our study presents new direct estimates of fine‐root productivity and turnover in a Central Amazonian plateau tropical forest, as well as the factors controlling their dynamics, which are crucial to the understanding of above‐ versus below ground trade‐offs and linkages determining forest function. We show that fine‐root productivity and mortality in surface layers were positively related to precipitation. Moreover, our results suggest a key interaction between water and nutrient availability affecting fine‐root productivity rates at our low soil P site. Fine‐root stock was greater in dry periods in the deepest layer where water is likely more available at that time. Fine‐root turnover decreased with soil depth, which can also have important implications for the soil carbon stock and the C cycling. Our results highlight the important role of deeper roots, with almost half of fine‐root productivity found below 30 cm soil depth. Fine‐root dynamics measurements are generally missing from deeper soil, which can lead to a misinterpretation of results from fine‐root dynamics in response to environmental changes. Therefore, depths below 30 cm should be taken into account as a standard procedure. Additional studies using minirhizotron estimates of deeper soil layers are highly recommended in different regions of the tropics to better understand how fine‐root dynamics and consequently the C cycle might change under disturbance. Our results, when considered with other minirhizotron studies, directly contribute to modeling projections for the future of tropical forests (Fleischer et al., 2019) and carbon storage, but uncertainties remain for how the precipitation regimes of low soil P tropical forest ecosystems will respond to global changes.

## CONFLICT OF INTEREST

The authors declare no conflict of interest.

[Correction added on 24 May 2021, after first online publication: Conflict of Interest statement added to provide full transparency.]

## AUTHOR CONTRIBUTIONS

A.L.C. collected and analyzed the data and wrote the manuscript. R.J.N., C.A.Q., I.P.H., D.M.L, and C.M.I. designed the study. E.O., N.B.G., B.T., C.M.I, and R.J.N. assisted in the field. R.J.N., C.A.Q., I.P.H., D.M.L., K.M.A., O.V‐B., L.F., N.B.G, and C.M.I. assisted with data analysis and contributed to the manuscript. All authors read and approved the manuscript.

## Data Availability

Data are freely available from the NGEE‐Tropics data archive: http://dx.doi.org/10.15486/ngt/1523508
