# Supplementary material for: Fine‐root dynamics vary with soil depth and precipitation in a low‐nutrient tropical forest in the Central Amazonia
Source: Plant Environ Interact. 2020 Apr 22;1(1):3–16. doi: 10.1002/pei3.10010 (PMC10168058; doi:10.1002/pei3.10010)
Supplement: Supplementary file 1 [file PEI3-1-3-s001.docx]

## *Plant-Environment Interactions* Supporting Information

Article title: **Fine-root dynamics vary with soil depth and precipitation in a low nutrient tropical forest in the Central Amazonia**

**Authors:** Amanda Longhi Cordeiro^1,2*^, Richard J. Norby^3^, Kelly M Andersen^4^, Oscar Valverde-Barrantes^1,5^, Lucia Fuchslueger^1,6^, Erick Oblitas^1^, Iain P Hartley^7^, Colleen M. Iversen^3^, Nathan B. Gonçalves^1,9^, Bruno Takeshi^1^, David M. Lapola^8^, Carlos Alberto Quesada^1^

1. Instituto Nacional de Pesquisas da Amazônia - INPA, Manaus, Brazil

2. Colorado State University – CSU, Fort Collins, United States

3. Oak Ridge National Laboratory, Tennessee, United States

4. Nanyang Technological University – NTU, Singapore

5. Florida International University - Miami, Florida, United States

6*.* University of Antwerp - Antwerp, Belgium

7. Geography, College of Life and Environmental Sciences, University of Exeter - UK

8. University of Campinas – UNICAMP, Campinas, Brazil

9. Michigan State University – MSU, East Lansing, United States

*Corresponding author, e-mail: [alonghicordeiro@gmail.com](mailto:alonghicordeiro@gmail.com); phone: +1 310 367 7940

The following Supporting Information is available for this article:

**Table S1:** Soil characteristics of the top 100 cm of the soil in the north-south transect adjacent to our plots.

| Depth (cm) | Ca | Mg | K | Na | Al | SB | ECEC | N | C | C/N |
| --- | --- | --- | --- | --- | --- | --- | --- | --- | --- | --- |
|  | cmolc kg^-1^ | | | | | | | % | % |  |
| 0-5 | 0.24 ± 0.02 | 0.30 ± 0.09 | 0.66 ± 0.7 | 0.22 ± 0.02 | 1.81 ± 0.24 | 1.42 ± 0.71 | 3.23 ± 0.49 | 0.26 ± 0.03 | 3.20 ± 0.31 | 12.17 |
| 5-10 | 0.07 ± 0.02 | 0.12 ± 0.03 | 0.08 ± 0.01 | 0.11 ± 0.04 | 1.88 ± 0.12 | 0.38 ± 0.1 | 2.25 ± 0.16 | 0.17 ± 0.01 | 2.19 ± 0.17 | 12.57 |
| 10-20 | 0.06 ± 0.01 | 0.06 ± 0.03 | 0.04 ± 0.01 | 0.04 ± 0.01 | 1.40 ± 0.21 | 0.20 ± 0.05 | 1.60 ± 0.25 | 0.12 ± 0.03 | 1.28 ± 0.28 | 11.21 |
| 20-30 | 0.07 ± 0.02 | 0.03 ± 0.01 | 0.02 ± 0.01 | 0.06 ± 0.03 | 1.12 ± 0.09 | 0.18 ± 0.03 | 1.30 ± 0.11 | 0.08 ± 0.01 | 0.85 ± 0.16 | 10.48 |
| 30-50 | 0.06 ± 0.01 | 0.02 ± 0.0 | 0.01 ± 0.0 | 0.03 ± 0.0 | 1.00 ± 0.11 | 0.13 ± 0.01 | 1.13 ± 0.11 | 0.06 ± 0.01 | 0.55 ± 0.08 | 9.36 |
| 50-100 | 0.06 ± 0.0 | 0.02 ± 0.01 | 0.01 ± 0.0 | 0.03 ± 0.0 | 0.99 ± 0.09 | 0.12 ± 0.02 | 1.12 ± 0.09 | 0.05 ± 0.01 | 0.44 ± 0.07 | 9.78 |

Soil measurements reported here were analyzed according to Quesada et al (2010). These soils were collected in three trenches at 20, 313 and 585 m across the north-south transect on the same plateau adjacent to our plots. Values represent mean ± standard error from those three soils.

**Table S2:** Models (lme or lm); dependent variable; fixed effect; random effect (if mixed linear model was performed); F and P value for mixed models and P value for linear regression model; and *r*^2^.

| **Model (lme)** | **Dependent variable** | **Fixed effect** | **Random effect** | **Weights** | **F and P value** | ***r*^2^** |
| --- | --- | --- | --- | --- | --- | --- |
| log(productivity) ~ depth_intervals | Fine-root productivity | Depth intervals (30 cm) | plot/tube | Depth intervals | (F_2, 16_ = 12.93, *P <* 0.001) | 0.84 |
| log(productivity) ~ season | Fine-root productivity | Season | plot/tube | - | (F_1, 8_ = 27.69, P < 0.001) | 0.66 |
|  |  |  |  |  |  |  |
| log(productivity) ~ diameter_classes | Fine-root productivity | Diameter classes | plot/tube |  | (F_3, 10_ = 23.33, *P <* 0.001) | 0.8 |
| log(productivity) ~ precipitation + depth_intervals | Fine-root productivity | Precipitation | time | - | (F_1,9_ = 25.06, *P <* 0.001) | 0.61 |
|  |  | Depth intervals (30 cm) | time |  | (F_2,18_ = 13.15, *P <* 0.001) |  |
| log(mortality) ~ depth_intervals | Fine-root mortality | Depth intervals (30 cm) | plot/tube | Depth intervals | (F_2, 16_ = 9.19, *P <* 0.003) | 0.85 |
| log(mortality) ~ season | Fine-root mortality | Season | plot/tube | - | (F_1, 8_ = 12.00, P < 0.009) | 0.5 |
|  |  |  |  |  |  |  |
| **Model (lme)** | **Dependent variable** | **Fixed effect** | **Random effect** | **Weights** | **F and P value** | ***r*^2^** |
| log(mortality) ~ diameter_classes | Fine-root mortality | Diameter classes | plot/tube | - | (F_3, 10_ = 9.19, *P <* 0.004) | 0.64 |
| log(mortality) ~ depth_intervals * precipitation | Fine-root mortality | Precipitation | time | - | (F_1, 9_ = 0.33, *P >* 0.5) | 0.64 |
|  |  | Depth intervals (30 cm) | time |  | (F_2, 14_ = 9.60, *P <* 0.03) |  |
|  |  | Precipitation * depth intervals (30 cm) | time |  | (F_2, 14_ = 6.47, *P <* 0.02) |  |
| log(stock) ~ depth_intervals | Fine-root standing stock | Depth intervals (30 cm) | plot/tube | Depth intervals | (F_2, 16_ = 6.88, P < 0.008) | 0.4 |
| log(stock) ~ season | Fine-root standing stock | Season | plot/tube | - | (F_1, 8_ = 4.76, P > 0.06) | 0.95 |
|  |  |  |  |  |  |  |
| log(stock) ~ diameter_classes | Fine-root standing stock | Diameter classes | plot/tube | - | (F_3, 11_ = 41.78, *P <* 0.001) | 0.87 |
| log(stock) ~ depth_intervals + precipitation | Fine-root standing stock | Precipitation | time | - | (F_1, 9_ = 7.09, *P <* 0.03) | 0.98 |
|  |  | Depth intervals (30 cm) | time |  | (F_2, 20_ = 18.69.16, *P <* 0.001) |  |
| log(turnover) ~ factor(depth_intervals) | Turnover | Depth intervals (30 cm) | plot | - | (F_2, 14_ = 4.86, *P <* 0.003) | 0.41 |
| log(turnover) ~ factor(diameter_classes) | Turnover | Diameter classes | plot/tube | Diameter classes | (F_2, 10_ = 9.41, *P <* 0.001) | 0.31 |
| **Model (lm)** | **Dependent variable** | **Fixed effect** | **Random effect** | **Weights** | **P value** | ***r*^2^** |
| turnover ~ depth_intervals | Turnover | Depth intervals (10 cm) | - | - | *P* < 0.01 | 0.62 |
